# Chronic Care in Primary Care: Exploring the Role and Impact of General Practice Pharmacists in Managing Long-Term Conditions in Northern Ireland

**DOI:** 10.3390/ijerph22020292

**Published:** 2025-02-16

**Authors:** Ahmed Abuelhana, Petra Garlone Clark, Aaron Courtenay, Heather Coleman, Nermeen Ali, Kingston Rajiah

**Affiliations:** School of Pharmacy and Pharmaceutical Sciences, Ulster University, Coleraine BT 52 1SA, UK; a.abuelhana@ulster.ac.uk (A.A.); clark-p@ulster.ac.uk (P.G.C.); a.courtenay@ulster.ac.uk (A.C.); h.coleman@ulster.ac.uk (H.C.); n.ali@ulster.ac.uk (N.A.)

**Keywords:** general practice pharmacists, chronic conditions, primary care, role clarity, clinical confidence, mixed-methods study, Northern Ireland, medications

## Abstract

The role of General Practice Pharmacists (GPPs) has expanded in response to increasing demands on primary care services, particularly in managing chronic conditions. While GPPs are recognised for their contributions to medication optimisation and patient care, challenges such as role clarity, workload pressures, and confidence in clinical decision-making remain underexplored. This study aims to investigate the tasks, professional identity, confidence levels, and challenges faced by GPPs in Northern Ireland. A mixed-methods design was employed, incorporating a questionnaire distributed to GPPs across Northern Ireland. The questionnaire comprised 20 multiple-choice questions and 5 open-ended questions, focusing on demographics, tasks, confidence levels, role clarity, and professional challenges. Quantitative data were analysed using descriptive and inferential statistics, while qualitative responses underwent thematic analysis using NVIVO software. A total of 44 GPPs participated, with a majority being female and aged 34–39 years. Most participants had 4–6 years of experience as GPPs. Quantitative findings revealed significant correlations between clinical confidence and factors such as years of experience, age, and employment type. Qualitative analysis revealed key themes: clinical confidence was enhanced by training and experience, but workload pressures often limited time for patient care. Variability in role integration and the lack of public awareness were highlighted as barriers to maximising the GPP role. This study highlights the key challenges of workload distribution and role ambiguity in the GPP role. Delegating administrative tasks and developing clear frameworks for role integration could address these barriers. Additionally, targeted training programs and public education campaigns are essential to enhance the impact of GPPs in primary care.

## 1. Introduction

The increasing prevalence of multimorbidity and chronic conditions, particularly among the ageing population, has placed significant strain on healthcare systems worldwide [1]. In the UK, primary care services are under immense pressure due to the growing demand for chronic disease management and workforce shortages, particularly among General Practitioners (GPs) [2]. This has led to reduced job satisfaction, a decreased quality of care, and a rising burden on GPs who are tasked with managing an increasing number of complex cases. As healthcare evolves, there is a need for innovative solutions to address these challenges. One such solution is the role of the General Practice Pharmacist (GPP), introduced in Northern Ireland in 2015 as part of a broader initiative to enhance primary care services. By 2020, the service was fully implemented, and today, every GP practice in Northern Ireland has a pharmacist as an integral member of the clinical team [3]. GPPs undergo additional training beyond standard pharmacy qualifications, including primary care training programs, independent prescribing (IP) certification, and advanced clinical skills development. While no single mandatory exam exists for GPPs, structured training pathways, such as Centre for Pharmacy Postgraduate Education (CPPE) programs and equivalent regional schemes, ensure their integration into GP practices, enhancing their role in chronic disease management and medication optimisation. The GPP role was designed to address the growing need for comprehensive medication management, chronic disease monitoring, and interprofessional collaboration within primary care settings [4]. By providing expert clinical support in medication optimisation and patient education, GPPs can alleviate some of the pressure on GPs, enabling them to focus on more complex cases while improving patient outcomes in the management of long-term conditions.

The integration of GPPs into multidisciplinary teams (MDTs) has been shown to foster better collaboration and streamline care delivery, benefiting both patients and healthcare professionals [5]. GPPs contribute specialised medication expertise, support patient-centred care, and facilitate better clinical decision-making, ultimately improving patient outcomes and team efficiency. Despite the potential benefits of the GPP role, there remains limited research specifically exploring its impact on chronic disease management in Northern Ireland. While studies have demonstrated that pharmacists can play a vital role in enhancing medication adherence, optimising treatment plans, and reducing hospital admissions [6,7], there is a gap in understanding how GPPs contribute to the management of chronic conditions in primary care settings. Moreover, little is known about how GPPs perceive their role within GP practices, the challenges they face, and the interprofessional dynamics at play. These insights are essential for maximising the effectiveness of the GPP role and improving patient outcomes.

The rising prevalence of chronic conditions, such as cardiovascular diseases, diabetes, and respiratory disorders, is responsible for a significant proportion of healthcare expenditure [8]. These conditions require ongoing management, making primary care the focal point for patient care. Identifying the barriers that limit GPPs’ potential and the support they need to enhance their impact on patient outcomes is critical. To address this research gap, this study will explore how GPPs perceive their role in managing chronic conditions, assess their integration into GP practices, and examine how they contribute to improving patient care, optimising medication use, and fostering collaboration within primary care teams in Northern Ireland.

## 2. Materials and Methods

This study employed convergent parallel mixed-methods designs, integrating both quantitative and qualitative approaches to explore the role of GPPs in managing chronic conditions in Northern Ireland. The quantitative component aimed to quantify GPPs’ perceptions and experiences, while the qualitative component sought to gather in-depth insights into their views and the challenges they face in primary care settings.

### 2.1. Study Design

As of 31 March 2023, there were 317 active GP practices in Northern Ireland, with the total number of GPPs estimated to be equivalent to this number. However, specific data on the total number of pharmacists currently working within GP practices across Northern Ireland are not readily available. For this study, the distribution of the questionnaire was facilitated through the GPP federation, extending to all GPPs across the six counties in Northern Ireland.

### 2.2. Sampling Method

Purposeful sampling was employed to select GPPs with at least 2 years of experience working within GP practices in Northern Ireland. This sampling approach ensures that only those GPPs who meet the experience criteria are included, enabling the data collected to be representative of pharmacists working across the region with significant exposure to the role. This method allowed for the inclusion of GPPs who can offer informed perspectives on the challenges and practices related to their work in GP settings. Pharmacists working in other sectors of pharmacy (such as hospital or community pharmacy) were excluded from participating to ensure the focus remains on those within the primary care sector where the study’s objectives are centred.

### 2.3. Ethics and Approval

The study received ethical approval from the School of Biomedical Sciences Ethics Filter Committee at Ulster University (reference number FCBMS-22-116). Ethical considerations were addressed by ensuring that participants were fully informed of the study’s purpose, procedures, and their rights as participants, including the right to withdraw at any time without consequence. The data were stored securely, and participant anonymity was maintained throughout the study.

### 2.4. Questionnaire

The questionnaire was designed to collect both quantitative and qualitative insights, offering a comprehensive exploration of the GPP role. It consisted of four sections with a total of 20 questions (Refer Supplementary Materials). Section 1 focused on demographic information, comprising five items. Section 2 assessed confidence levels with one item, while Section 3 gathered information on tasks and responsibilities through one item. The final section included 13 open-ended questions, which were designed to capture qualitative data, providing a more in-depth understanding of the GPP’s role. The questionnaire was piloted with five GPPs, and changes were made based on feedback to ensure clarity, relevance, and alignment with the study objectives. The piloted data were not included in the study.

### 2.5. Data Collection Procedures

Anonymous data were collected using the Jisc^®^ online survey platform. Participation was voluntary, and informed consent was clicked electronically by all participants prior to completing the survey. Participants were assured of the confidentiality of their responses and that the data would be used solely for this study.

### 2.6. Data Analysis

Quantitative data were analysed using SPSS^®^ software version 22. Descriptive statistics were used to summarise participant demographics and responses to structured questions. To determine the relationships between variables, the Pearson Chi-squared test was applied to assess associations between GPPs’ demographic characteristics and their confidence. A significance level of *p* < 0.05 was considered statistically significant.

For qualitative data, thematic analysis was performed to explore the key issues raised by participants, including their perceptions of their role within MDTs, challenges in clinical decision-making, and the support needed for effective chronic disease management. NVIVO was used to code the responses and categorise them into thematic areas, providing a rich narrative of GPPs’ experiences.

## 3. Results

### 3.1. Quantitative Findings

A total of 44 GPPs participated in the study, representing all six counties of Northern Ireland. The sample was predominantly female, with the largest age group falling between 34 and 39 years. Most participants had 4–6 years of experience in the GPP role, and most of the participants were working full-time. Table 1 summarises the demographic characteristics of the participants.

**Figure 1:**
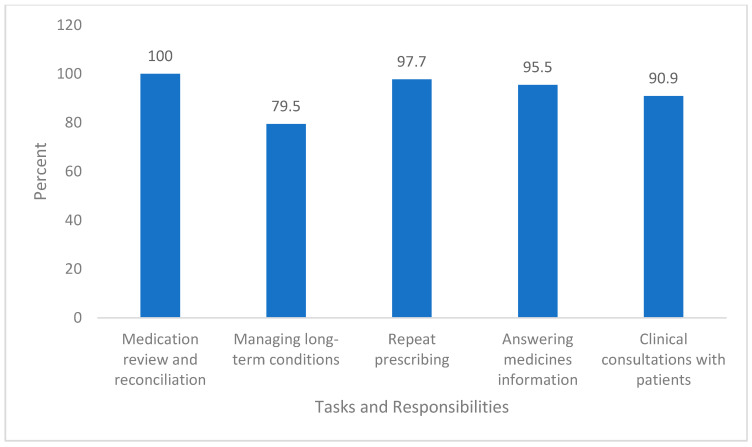
Tasks and responsibilities performed by participants.

Participants were asked about the tasks they performed in their role as GPPs. All of them reported conducting medication reviews and reconciliations. The most commonly managed chronic conditions were cardiovascular diseases (68.2%), followed by respiratory conditions, including asthma and COPD (54.5%). Other common responsibilities included managing repeat prescriptions (97.7%) and answering medicine-related queries (95.5%). Figure 1 shows the distribution of tasks carried out by GPPs.

Table 2 represents the relationship between demographic characteristics and confidence levels of the participants. Age positively influenced confidence, with GPPs aged over 45 years reporting the highest confidence levels (*p* = 0.031, χ^2^ = 7.91). Confidence increased with years of experience as a GPP, with those having 10 or more years of experience in the role reporting the highest levels of confidence (*p* = 0.024, χ^2^ = 13.78). Employment type was another significant factor, with full-time GPPs exhibiting higher confidence than part-time GPPs (*p* = 0.043, χ^2^ = 9.34). Gender differences in confidence levels were not statistically significant.

### 3.2. Qualitative Findings

Table 3 summarises the themes and sub-themes of this study.

#### 3.2.1. Professional Identity and Role Clarity

A clear professional identity was perceived as important for GPPs. While 84.1% of participants expressed having a clear understanding of their role, 15.9% reported uncertainty, particularly regarding the scope of responsibilities. Many GPPs highlighted the need for clearer frameworks defining their tasks and responsibilities within the MDT.

Supporting quotation: “*I feel I have a strong professional identity as a pharmacist, but there’s still confusion about the scope of my role within the GP practice. More clarity on my duties would help.*”

#### 3.2.2. Interprofessional Collaboration

The quality of collaboration between GPPs and other healthcare professionals was generally positive, with 68.2% of participants reporting that their relationship with GPs was “very good” or “good”. However, some GPPs noted variability in support across practices, with more established roles and longer working relationships leading to better integration within the team.

Supporting quotation: “*In some practices, the collaboration with GPs is fantastic. However, in others, there’s still a reluctance to fully integrate GPPs into the decision-making process*.”

#### 3.2.3. Training and Support Needs

Many GPPs expressed the need for additional training, especially in clinical decision-making for chronic conditions such as diabetes, hypertension, and asthma. While the majority of participants reported receiving support from their colleagues, 22.7% noted insufficient time for training, particularly in clinical skills like interpreting blood results and spirometry.

Supporting quotation: “*More training on interpreting blood tests and spirometry would help me feel more confident in managing chronic conditions*.”

#### 3.2.4. Public Awareness of the GPP Role

A significant barrier identified by GPPs was the lack of public awareness of their role as independent prescribers. In total, 88.6% of participants felt that patients were unaware of the GPP role, with many expressing that patients often mistook them for community pharmacists rather than primary care clinicians.

Supporting quotation: “*Patients are often confused and think I’m just a community pharmacist. They don’t realise I can prescribe and manage their chronic conditions*.”

#### 3.2.5. Clinical Confidence

Participants highlighted varying levels of confidence in managing chronic conditions, which may be influenced primarily by their years of experience and the availability of clinical training. More experienced GPPs reported higher confidence in handling complex cases, interpreting diagnostic tests, and making independent clinical decisions. However, less experienced GPPs, especially those with under 3 years of practice, indicated uncertainty in managing multimorbidity and interpreting specific clinical results like blood work and spirometry.

Supporting quotations: “*With over 10 years in practice, I feel confident in making decisions for diabetes and hypertension patients. Training helped reinforce this confidence*.”

“*I often doubt my abilities when it comes to interpreting complex diagnostic results. Additional training would really help.*”

#### 3.2.6. Workload

Workload emerged as a consistent theme, with many GPPs describing their responsibilities as extensive and, at times, overwhelming. Tasks such as repeat-prescribing and answering medicine-related queries were reported to consume a significant portion of their time, often limiting opportunities for patient consultations and chronic disease management. Some participants expressed concern about the balance between administrative duties and clinical responsibilities.

Supporting quotations: “*The workload is immense. Sometimes it feels like I’m doing more paperwork than managing patients*.” “*Repeat prescribing takes up most of my day, leaving little time for more meaningful clinical work with patients*.”

#### 3.2.7. Role Integration

While many GPPs felt integrated into their MDTs, others reported variability in the level of collaboration and recognition within different practices. Established relationships with GPs and nurses often facilitated better integration, whereas, in some settings, GPPs felt underutilised or unclear about their responsibilities. Participants highlighted the need for clearer role definitions and greater support from healthcare leadership to ensure their clinical skills were fully utilised.

Supporting quotations: “*I have a strong relationship with my GP colleagues, and we work well together on chronic care*.” “*In some practices, I feel like I’m just doing tasks rather than being part of the team. There’s a lack of clarity about my role*.”

## 4. Discussion

This study explored the role of GPPs in managing chronic conditions in Northern Ireland, offering valuable insights into their professional identity, tasks, confidence levels, and challenges in primary care. The findings highlight context-specific issues that warrant attention to maximise the impact of GPPs in primary care settings.

The demographic profile of participants reflects broader trends in the pharmacy profession, where women constitute the majority of practising pharmacists [9]. Most GPPs in this study were 34–39, suggesting that the role often attracts experienced pharmacists transitioning into primary care. The relatively young median age of GPPs with 4–6 years of role-specific experience highlights the evolving nature of the GPP role in Northern Ireland, which was introduced as part of efforts to address workforce shortages and improve chronic disease management [10]. These findings align with trends showing a growing movement of pharmacists into primary care to address increasing healthcare demands [11].

The study highlights the broad scope of responsibilities undertaken by GPPs, ranging from medication reviews to managing chronic conditions and conducting patient consultations. The finding that all participants conducted medication reviews aligns with existing research emphasising this as a cornerstone of the GPP role [12,13]. These medication reviews optimise pharmacotherapy and enhance patient safety, particularly for those with complex medication regimens [14]. The high prevalence of tasks such as repeat-prescribing and addressing medication-related queries highlights the critical role GPPs play in ensuring continuity of care for long-term conditions [15]. This kind of work by GPPs reduces GP workload while improving the quality of care [16]. Chronic conditions most commonly managed by GPPs, such as cardiovascular diseases and diabetes, reflect global trends in pharmacy practice [17].

This study reinforces the link between experience and confidence in clinical decision-making by pharmacists [18]. GPPs with 10 or more years of experience reported the highest levels of confidence, suggesting that accumulated clinical exposure enables them to navigate complex cases and contribute more effectively to MDTs. Similarly, confidence increased with age, a trend observed across healthcare professions where maturity and professional longevity enhance self-assurance [19]. Full-time GPPs also reported higher confidence than their part-time counterparts, likely due to increased exposure to clinical scenarios and greater integration into team dynamics [20,21]. Addressing confidence gaps for part-time GPPs through targeted training and mentorship programs could mitigate these disparities and ensure equitable development opportunities. Gender did not significantly influence confidence levels reflecting an equitable working environment within Northern Ireland’s GPP framework, which supports professional growth across genders. International frameworks such as Canada’s Patient-Centred Care, Australia’s National Medicines Policy, and the U.S. Clinical Pharmacy Practice Model offer valuable insights into integrating pharmacists into healthcare teams. These models emphasise collaborative care, medication optimisation, and chronic disease management, which could be adapted to enhance the role of GPPs in Northern Ireland’s primary care system.

A recurring theme in the qualitative data was the need for clearer role definitions. While most participants felt they had a well-defined professional identity, some expressed uncertainty about their responsibilities within MDTs. This aligns with studies reporting that ambiguous role expectations can lead to underutilising pharmacists’ skills [22,23]. In this study, GPPs described their role as complex, encompassing medication reconciliation, patient education, and interprofessional collaboration. However, the lack of formalised frameworks for their responsibilities limits their ability to work to their full potential. Clearer role definitions and onboarding processes could enhance the integration of GPPs into MDTs, fostering their contributions to chronic disease management.

The study highlights a strong need for additional training, particularly in clinical skills such as interpreting blood tests and spirometry. Many participants felt that time constraints in primary care limited opportunities for professional development. This emphasises that ongoing training is essential for pharmacists to manage the complexities of multimorbidity effectively [24]. While GPPs in Northern Ireland do receive training in chronic disease management, the study findings suggest that ongoing development is necessary, particularly in diagnostic skills, evolving guidelines, and clinical decision-making confidence. Compared to countries like Canada and the USA, where structured postgraduate clinical training is common before pharmacists enter primary care, Northern Ireland’s model places GPPs into primary care roles sooner, which may explain some of the reported gaps in confidence. Investing in structured, hands-on clinical training such as supervised case management, diagnostic skill-building, and interprofessional mentorship could bridge this gap and further enhance GPPs’ contributions to chronic disease management. This recommends the importance of equipping pharmacists with advanced skills to meet the demands of modern primary care [25].

Workload emerged as a significant barrier, with GPPs reporting that routine administrative tasks often overshadowed opportunities for direct patient care. This aligns with a report that noted an excessive administrative burden can hinder pharmacists from fully utilising their clinical skills [26]. Reducing non-clinical duties by delegating tasks such as repeat-prescribing to administrative staff or pharmacy technicians could free up GPPs to focus on patient-centred activities. This would improve job satisfaction and enable GPPs to dedicate more time to managing chronic conditions, ultimately enhancing patient outcomes. The GPP workforce in Northern Ireland currently does not include pharmacy technicians, which sets it apart from the rest of the UK where pharmacy technicians are integral to the GPP model.

Variability in role integration across practices highlights a critical gap in consistency. While some GPPs reported positive relationships with GPs and nurses, others described frustration due to unclear responsibilities or limited collaboration. This variability reflects that inconsistent integration of pharmacists into MDTs can lead to the underutilisation of their expertise [27]. Clear frameworks and regular team meetings could help standardise the integration process, ensuring GPPs are fully embedded within primary care teams. Joint training sessions and case reviews could further strengthen interprofessional collaboration, enhancing team dynamics and mutual understanding of roles [28].

A significant barrier identified in this study was the lack of public awareness about the GPP role. Most participants reported that patients often confuse GPPs with community pharmacists, limiting the recognition of their clinical capabilities. This may affect patient engagement and understanding in optimising healthcare interventions. Public education campaigns highlighting the unique role of GPPs in managing chronic conditions could improve patient awareness and encourage a greater utilisation of their services. Such efforts would enhance the GPP’s impact and strengthen patient–pharmacist relationships and trust.

### 4.1. Implications for Practice and Policy

The findings of this study highlight several implications for practice and policy to enhance the impact of GPPs in primary care. Tailored training programs focusing on clinical skills such as diagnostic interpretation, chronic disease management, and multimorbidity are essential to address confidence gaps, particularly among less experienced and part-time GPPs. Strategies to manage workloads, including delegating non-clinical tasks like repeat-prescribing to administrative staff or pharmacy technicians, could enable GPPs to prioritise patient-centred care and optimise their clinical contributions. Pharmacy technicians in Northern Ireland are not yet a registered profession so proposed amendments to the Medicines Act and Human Medicines Regulations for registered pharmacy technicians do not apply. The Department of Health, Northern Ireland, is consulting on regulating pharmacy technicians to align with the rest of the UK. Role clarity remains a critical area, requiring the development of standardised frameworks to define GPP responsibilities and ensure consistent integration into MDTs. Primary care teams should establish formal induction programs where new GPPs, GPs, and nurses align expectations early. Encourage joint clinical case reviews, shared decision-making meetings, and shadowing opportunities between GPPs and other healthcare professionals. Additionally, public awareness campaigns should be launched to educate patients about the clinical capabilities of GPPs, fostering trust and encouraging the utilisation of their services. These measures would strengthen the role of GPPs, improving chronic disease management and alleviating pressure on primary care teams.

### 4.2. Limitations

The sample size may not fully represent the broader population of GPPs across Northern Ireland. Since the study relies on purposeful sampling, which targets GPPs with at least 2 years of experience within GP practices, it is possible that the sample may not reflect the full range of experiences or perspectives of GPPs, especially those with less than 2 years of experience. Though 44 GPPs have responded to the questionnaire, the total number of GPPs with at least 2 years of experience is unknown, as is the number of GPPs who have not responded. This limits our ability to assess the representativeness of the sample and may affect the generalisability of the findings to the entire GPP population in Northern Ireland. The sampling method may have excluded some non-Federated GPPs, which could impact the representativeness of the sample. However, this limitation is mitigated by the focus on experienced practitioners, whose insights are critical to the research.

## 5. Conclusions

This study highlights the critical role of GPPs in enhancing the management of chronic conditions within primary care. The findings highlight the importance of role clarity, public awareness, and adequate training to maximise the impact of GPPs. While many GPPs feel confident in their ability to manage chronic conditions, there are notable gaps in clinical training and variability in their integration across practices. Addressing these barriers is crucial for ensuring that GPPs can fulfil their potential as key contributors to primary care teams. By optimising medication therapy, providing personalised patient support, and collaborating with MDTs, GPPs alleviate the burden on GPs and contribute to improved patient outcomes. Their integration into primary care is particularly relevant in addressing the growing prevalence of multimorbidity and the increasing complexity of chronic disease management.

## Figures and Tables

**Table 1:** Demographic characteristics of participants.

Demographic Characteristic	Number (n = 44)	Percentage (%)
Gender		
Female	32	72.7
Male	12	27.3
Age Group		
28–33 years	10	22.7
34–39 years	20	46.3
40–45 years	7	16.0
>45 years	7	16.0
Years of Experience as a Pharmacist Before GPP		
1–2 years	4	9.1
3–5 years	25	56.8
6 years or more	15	34.1
Years of Experience as a GPP		
1–3 years	4	9.1
4–6 years	25	56.8
7–9 years	10	22.7
10 years or more	5	11.4
Employment Type		
Full-time	26	59.1
Part-time	18	40.9
County		
Derry/Londonderry	2	4.3
Antrim	18	41.3
Tyrone	8	17.4
Fermanagh	3	6.5
Down	5	10.9
Armagh	9	19.6

**Table 2:** Relationship between demographic characteristics and confidence levels.

Demographic Characteristic	Very Confident (%)	Confident (%)	Not Confident (%)	Not Very Confident (%)	Chi-Square Values (χ^2^ Statistics)	*p*-Value
Gender						
Male	25	50	25	0	0.87	0.164
Female	15	58	23	4		
Age Group						
28–33 years	10	40	40	10	7.91	0.031 *
34–39 years	20	60	20	0		
40–45 years	30	50	20	0		
>45 years	40	50	10	0		
Years of Experience as GPP						
1–3 years	10	35	45	10	13.78	0.024 *
4–6 years	20	55	20	5		
7–9 years	35	50	15	0		
10 years or more	50	40	10	0		
Employment Type						
Full-Time	25	55	15	5	9.34	0.043 *
Part-Time	10	50	35	5		

* *p* < 0.05.

**Table 3:** Themes and sub-themes.

Theme	Sub-Theme	Representative Quotations
Professional Identity and Role Clarity	Role Ambiguity	“*I have a strong professional identity, but there’s confusion about the scope of my role.*”
	Role Clarity	“*The more established I become, the clearer my responsibilities in the team are.*”
Interprofessional Collaboration	Positive Collaboration	“*In some practices, the collaboration with GPs is fantastic; we work really well together.*”
	Variability in Support	“*Some practices are more supportive than others. I often feel like the role is underutilized in certain settings.*”
Training and Development	Need for Clinical Training and More Opportunities	“*I feel unprepared when it comes to interpreting blood results and spirometry.*” “*We often don’t have enough time for training, especially with the time constraints in primary care.*”
Public Awareness of the GPP Role	Low Awareness among Patients	“*Patients often think I’m a community pharmacist and don’t realize I can prescribe and manage their chronic conditions.*”
	Need for Public Education	“*There needs to be more awareness campaigns about what we can do for patients, especially regarding chronic condition care.*”
Clinical Confidence	Confidence in Decision-Making and Areas of Uncertainty	“*I feel confident in managing hypertension and diabetes, especially after years of experience.*” “*I still lack confidence when making decisions for complex cases like COPD or managing multiple comorbidities.*”
Workload and Role Integration	Task Overload	“*There is a lot of pressure to take on routine tasks like repeat prescriptions, which sometimes limits time for complex care.*”
	Underutilisation of Clinical Skills	“*I often feel that the full range of my skills isn’t being used, particularly in terms of managing chronic diseases.*”

## Data Availability

Data presented in the study are stored securely at school of Pharmacy and Pharmaceutical Sciences, Ulster University. Investigators act as custodians for the data processed and generated by the study and they are also responsible for the access to any information included. Data are available upon request from the corresponding author. Due to privacy and institutional regulations, the data are not publicly accessible.

## Supplementary Materials

The following supporting information can be downloaded at: https://www.mdpi.com/article/10.3390/ijerph22020292/s1, File S1: Questionnaire.
