# Supplementary material for: Chronic Care in Primary Care: Exploring the Role and Impact of General Practice Pharmacists in Managing Long-Term Conditions in Northern Ireland
Source: Int J Environ Res Public Health. 2025 Feb 16;22(2):292. doi: 10.3390/ijerph22020292 (PMC11855525; doi:10.3390/ijerph22020292)
Supplement: Supplementary file 1 [file ijerph-22-00292-s001.zip › ijerph-3429117-supplementary.pdf]

Questionnaire

Section 1: Demographic Information

1. What is your gender?
    - Male
    - Female
    - Prefer not to say
2. What is your age group?
    - 28–33 years
    - 34–39 years
    - 40–45 years
    - 45 years
3. How many years of experience do you have as a General Practice Pharmacist (GPP)?
    - 1–3 years
    - 4–6 years
    - 7–9 years
    - 10 years and more
4. What is your current employment type?
    - Full-time
    - Part-time
5. In which county are you working?
    - Derry/Londonderry
    - Antrim
    - Tyrone
    - Fermanagh
    - Down
    - Armagh

Section 2: Confidence Levels

6. How confident do you feel in making clinical decisions for patients with chronic conditions?
    - Very confident
    - Confident
    - Neutral
    - Not confident

Section 3: Tasks and Responsibilities

7. Which tasks do you regularly perform as part of your role? (Select all that apply)
    - Medication reviews
    - Managing repeat prescriptions
    - Answering medicine-related queries
    - Managing chronic conditions
    - Running patient consultations
    - Other (please specify): ___________

Section 4: Qualitative Questions

8. How would you describe the quality of collaboration between you and other healthcare professionals? Are there any areas for improvement?
9. Which aspects of your role as a GPP are clearly defined within the multidisciplinary team? What areas require more clarity or better definition?
10. What specific training have you received to effectively manage chronic conditions? How has this training prepared you for your role?
11. Which areas of training do you feel need further development to enhance your confidence and effectiveness as a GPP?
12. How do you think patients perceive and understand your role as a GPP in managing their health? Are there any misconceptions?
13. What strategies or initiatives do you believe would help improve public awareness of the GPP role and its importance in healthcare?

14. What are the most significant challenges you face in your role as a GPP, particularly when managing chronic conditions?
15. What factors or resources have been most helpful in enabling your success as a GPP?
16. How do you think the GPP role could be better supported or integrated into primary care teams to improve collaboration and patient outcomes?
17. What specific changes, initiatives, or resources do you believe would enhance the impact of GPPs in managing chronic conditions?
18. Can you share a specific example where your role as a GPP significantly improved patient outcomes or contributed to better healthcare delivery?
19. How do you typically communicate with other healthcare professionals about patient care, and what feedback mechanisms are in place to ensure effective collaboration?
20. How do you ensure that your approach as a GPP aligns with patient-centred care principles, particularly when managing chronic conditions?
